# Anti-Racist Practices in Health Care Organizations—A Qualitative Analysis

**DOI:** 10.3390/ijerph22111641

**Published:** 2025-10-28

**Authors:** Sidra Khan-Gökkaya, Faye McMillan, David R. Williams

**Affiliations:** 1 Department of Social and Behavioral Sciences, Harvard T.H. Chan School of Public Health, Harvard University, Boston, MA 02115, USA; dwilliam@hsph.harvard.edu; 2 Department of Patient and Care Management, University Medical Center Hamburg-Eppendorf (UKE), Martinistraße 52, 20246 Hamburg, Germany; 3 Faculty of Health, Kuring-gai Campus, University of Technology Sydney, Sydney, NSW 2007, Australia; fmcmill1@jh.edu; 4 Department of African & African American Studies, Harvard University, Cambridge, MA 02138, USA

**Keywords:** racism, health care, organizations

## Abstract

Introduction: A considerable body of evidence shows significant racial inequities in health and health care, affecting access, care and treatment for patients, as well as the wellbeing of employees. Many hospitals and health care organizations have committed to anti-racist change within their systems. Still, there is limited systematic knowledge regarding organizational anti-racist practices, the conditions under which they can be implemented successfully and their effectiveness. This research aims to identify anti-racist practices within health care organizations with a special focus on three areas: (1) increasing workforce diversity, (2) reducing racial health disparities and (3) responding to discriminatory behavior. Moreover, the role of different stakeholders in implementing anti-racist change will be analyzed, as well as the challenges organizations have encountered and strategies they have utilized to implement change. Methods: Primary (*n* = 11) and secondary qualitative data (*n* = 26) were used to gain insights from anti-racism diversity experts and health equity officers within organizations across the US in the beginning of 2024. A qualitative content analysis was used to identify anti-racist practices in organizations. Results: Findings reveal a broad range of anti-racist practices in use across these organizations. These practices include (1) collecting patient and staff data, (2) actively normalizing and implementing anti-racist work standards and guidelines, (3) developing organizational policies and tools to address racism, (4) creating accountability procedures for addressing racist behavior and (5) building safe and culturally appropriate spaces for racialized communities. By embedding a structural anti-racist lens across these organizations, stakeholders acknowledge their role in (past) harms and commit to addressing disparities in health care and creating a vision for health equity. Conclusion: The identification of anti-racist practices makes solutions visible to a broader audience and identifies the potential influence and responsibility each stakeholder in health care has to address racism. In order to apply these practices to other health care organizations, there is a need to rigorously evaluate the interventions and analyze their effectiveness.

## 1. Introduction

“Racism is a public health crisis”. This was a declaration issued by many health care organizations in the US following policies and resolutions to address systemic racism in health care [1]. The Black Lives Matter Movement, as well as the COVID-19 pandemic, amplified the systemic and racial inequities in health care, leading to a shift toward more equity-oriented and anti-racist care. This research was conducted prior to recent shifts in the US policy and funding environment regarding diversity, equity, inclusion and anti-racism. These shifts and their implications for the findings are addressed in the Discussion Section.

Addressing racism in health care requires the implementation and application of strategies, policies and actions throughout the health care ecosystem and beyond. Evidence suggests the effectiveness of some federal- and state-level policies in reducing racial inequities in health care [2]. Moreover, researchers stress how important it is that “public health, medicine and arguably, science, must reconcile and commit to making meaningful change to address structural racism and the deepening health disparities. It [public health, medicine and science] has to re-examine the ways in which ‘evidence’ and ‘data’ are collected, what working with community organizations and residents will require to create new models of care and safety, and what we have to learn together to drive that change. Therefore, we believe accountability is that titration process” [3].

Apart from federal- and state-level stakeholders, one essential stakeholder within the health care ecosystem to hold accountable is health care organizations. While studies have indicated that there is an overemphasis on individual-level anti-bias training within health care [4,5], there is little evidence regarding specific practices that aim to address racism at the organizational level. Anti-bias training can increase awareness among health professionals, but it does not necessarily lead to a change in behavior [6]. Change at the individual level requires more intense engagement, such as 12 weeks of training and homework exercises [7] that may be difficult to incorporate and clearly highlighting the need for broader organizational changes. Thus, “organisations should avoid implementing stand-alone individual-level training and instead shift their focus and resources to policies and practices that seek to dismantle pervasive institutional and systemic racism through a multi-level approach” [4]. In recent years, organizations have acknowledged and begun shifting toward becoming anti-racist organizations as a result of broader macro political and population health events such as the Black Lives Matter Movement and the COVID-19 pandemic. Despite the policy shift, this field is relatively understudied [8], and understanding early efforts offers valuable insights for sustaining equity work over time. Thus, this study aims to address this gap by identifying anti-racist practices within organizations that aim to reduce racial disparities, increase workforce diversity and address racist behavior from patients or staff. Moreover, the study sheds light on the roles that different stakeholders have in the health care system that can further advance equity in health care. Building on Calliste and Dei’s definition of anti-racism, which frames anti-racism as an ‘action-oriented, educational and/or political strategy for systemic and political change that addresses issues of racism and interlocking systems of social oppression’ [9], we situate anti-racism within a structural racism framework. This definition is particularly aligned with the goals of our study, as it recognizes the multifaceted nature of anti-racism. Applied to health care organizations, this means creating accountability mechanism for addressing racist behavior, embedding equity goals into organizational structures and developing a workforce that counteracts disparities. To situate this study, we draw on existing frameworks that distinguish between individual-, organizational-, community- and system-level interventions to address racism in health care [4]. While anti-racism work should encompass strategies across all levels, this study focuses specifically on the organizational level, where practices such as accountability mechanisms, policies to address discrimination and workforce diversity are implemented. The organizational change model by Kotter [10] complements this by informing the implementation analysis. It provides the necessary tools to understand how institutions successfully adopt, implement and sustain anti-racist practices, emphasizing how institutions adopt, implement and sustain new anti-racist practices.

## 2. Materials and Methods

This study used primary qualitative data, as well as secondary qualitative data. Reporting the primary qualitative data follows the consolidated criteria for reporting qualitative research (COREQ) guidelines [11]. This study was reviewed by the Harvard T.H. Chan Faculty of Medicine Institutional Review Board (IRB24-0010) and received an exemption for human subject research on 10 January 2024 due to minimal risk for participants. In accordance with this decision, no intentional demographic data were collected in order to ensure the anonymity of the interviewees and their organizations.

Literature research and web searches were conducted to identify US-based experts on anti-racist change in health care for the primary dataset. To qualify for inclusion in this study, experts were required to have a minimum of 3 years of experience in the respective field and work in anti-racism, health equity or diversity manager positions within a health care organization in the US. An interview guideline was developed and tested prior to interviews with three colleagues (researchers and public health practitioners) working in the field of health equity, which led to minor adjustments in wording. The final interview guideline consisted of three sections: (1) the expert’s role within their organizations and anti-racist practices within their organizations; (2) their perspective on anti-racist change in health care, the role of each organization and the challenges and barriers they experienced working in their role; and (3) their vision for anti-racist change in health care. Our approach followed a purposive sampling strategy aimed at individuals with specific knowledge in this area who met the predefined criteria. Recruitment was conducted through professional networks, referrals and publicly available information, ensuring a range of organizations with different approaches to anti-racist change.

The 22 identified experts for the primary dataset were contacted by email, informed about the study prior to the interview and then invited to participate in an interview. No incentives were offered for participation. Eleven experts responded and agreed to participate. Response rates were influenced by factors such as time frames and availability during the timeframe of the research. Although individual demographic data were not collected, participants and non-participants were compared using available organizational characteristics (e.g., professional role, position level). On this basis, no substantial differences were evident between participants who refused and participated in the interviews. After obtaining consent, interviews were conducted online via Zoom and transcribed. The interviews (*n* = 11) lasted from 30 to 60 min. All interviews were conducted by the first author, a female postdoc researcher with extensive experience in conducting qualitative interviews. The first author’s background in migration studies and their anti-racism work in a European country has influenced the framing of this study, as well as the three focus areas of the interview. While the study was initially motivated by a practical interest in finding organizational solutions, the analysis was subsequently anchored in organizational change theory and a structural racism framework. To minimize bias, the researcher adhered strictly to the interview guideline and avoided deviations that could steer responses. The interviews were conducted between February 2024 and May 2024.

The secondary qualitative data are derived from a mixed-methods study addressing the work of health equity officers in the US and how hospitals address the effects of racism [12]. Health equity work was defined as any set of activities from designated health equity officers inside the hospital and in the community that aims to address health disparities and their underlying causes [12]. The purpose of this study was to examine the experiences of health equity officers in the US. In doing so, a structured survey and in-depth individual interviews were conducted. The secondary qualitative data are derived from this study and interviews with 26 health equity officers. A summary table of participant characteristics from the secondary dataset is provided in the Supplementary Material (File S1). The study had a separate ethical clearance from Mass General Brigham Institutional Review Board. In this study, the interview guideline asked for strategies, facilitators and barriers for engaging in health equity work, and one question explicitly addressed events of racism at their hospital. The authors used purposive sampling based on the experience of the health equity officer and the time they have worked in this position. Use of this data was carefully reviewed and discussed with qualitative research experts, and all transcripts (*n* = 26) were re-analyzed to ensure that they were of high quality and validity and that no data were overlooked. The total number of analyzed transcripts is 37, comprising 26 interviews from the secondary dataset and 11 interviews from the primary dataset (an overview of the methods is provided in the Supplemental Material/Table S1). All data were analyzed using content analysis [13] and the content analysis software MAXQDA 24.11 (a list of the coding tree is provided in the Supplemental Material (File S1)). To ensure reliability, the codes were reviewed and discussed among the first and second author until consensus was achieved. Based on the interview guidelines, we used a deductive approach for coding but also allowed inductive codes to emerge. By combining both primary and secondary data, we were able to triangulate our findings. The primary data provided us with specific examples of anti-racist practices and strategies used in health care organizations, while the secondary data offered broader system-wide trends.

## 3. Results

The analysis identified five key themes in anti-racist practices: (1) the need for engagement from all stakeholders in health care, (2) increasing workforce diversity and leadership representation, (3) addressing racial health disparities and other social drivers of health, (4) responding to racist behavior within organizations, and (5) recognizing challenges in anti-racism work and the strategies used to overcome them.

### 3.1. Embedding Anti-Racism Across All Levels of Health Care Organizations

The interviewees represented different sections of the health care ecosystem, including professional associations (PAs), city and state health departments (CSHDs), research institutions (RIs), philanthropic organizations (POs), community organizations (COs), major hospital systems and their health equity officers (HEQs). They all recognized their responsibility in addressing racism in health care and their influence to do so. “Just by naming what our role is, and being honest with ourselves and recognizing that it’s going to take every single one that’s part of that ecosystem to do their part, to do better” [CSHD, IP1]. Interviewees employed by research institutions saw their primary purpose as making data available and in turn bringing attention to the inequities in the system. They also felt that through the data, organizations would have the evidence to change the status quo in terms of the low rates of representation of Black, Indigenous and People of Color health professionals. Interviewees mentioned that they could use policies as levers to address change within their organization. In this context, they acknowledged the newly introduced equity goals through accreditation organizations, the required community health needs assessment and the equity measures introduced by the Centers for Medicare & Medicaid Services. Another source of influence identified was professional associations who provide roadmaps for promoting health equity within hospitals, advocate for large groups of professionals in health care and influence the ecosystem. This could be carried out by putting forward resolutions, such as “naming racism as a cause of inequities, ending racial essentialism and getting rid of race as a proxy in medicine” [PA, IP 4]. These resolutions provided a blueprint for other organizations to follow and use as guidance. Furthermore, these associations can “provide advocacy briefs in cases where anti-racism efforts were tried to be eliminated” [PA, IP 5] due to legal decisions. The associations also created peer network systems to install supporting structures for leaders regardless of their organizations or state’s commitment to anti-racism. Community organizations played an important role in providing culturally responsive and anti-racist care while also interacting with other stakeholders and advocating for patients’ rights. This was specifically pointed out for doulas and midwives around maternal health and birthing: “And also really being that face to interact with the medical system, right? Knowing all of the medical jargon, knowing what is happening during birth and to be able to advocate for the birthing person […] so that they don’t have to also be giving birth and advocating for their rights” [IP 11, CO].

Addressing racism in health care was also associated with philanthropic organizations and foundations, their grant-design and selection processes and the potential impact of this work: “we can use our power and our privilege and influence as a funder to actually hold healthcare institutions accountable” [PO, IP 7]. This came with a responsibility to critically self-reflect on their history and legacy in the context of racism, but also on their current procedures such as “who are the organisations we are working with? Are they led by communities most impacted? Who are the vendors we work with?” [PO, IP 7], and an obligation to tie support for organizations to having equity goals. This strategy was also used by state health departments, referring to policies requiring that “all state agencies who we have a contract with need to have a strategic plan on how to address health equity” [CSHD, IP 1], while also critically looking at their data and implementing targeted approaches to advance health equity. They used policies, such as bills, to co-create equity goals with different stakeholders, thereby connecting organizations on a meso and macro level. In doing so, they aimed to disrupt practices that pose a barrier to access to care and used these policies as a lever for change.

### 3.2. Increasing Workforce Diversity and Leadership Representation

There was consensus amongst all interviewees that the demographics of the workforce should represent the demographics of the communities they partner with and that broader efforts were needed to reach this aim. Interviewees talked about career development programs funded by hospitals that aimed to “bring people through an entire career progression” [HEQ, IP 9] and building career pathways for existing employees to improve retention rates. They talked of the importance of building partnerships with medical and nursing schools and recruiting minority fellows and residents, tailored programs to create a sense of belonging and providing career advancement opportunities to ensure their retention. Major strategies identified by interviewees for increasing workforce diversity were inclusive hiring practices such as using certain community channels to place job descriptions, targeted recruiting or asking potential [leadership] staff about their perspectives on inequities and racism in health care to make sure the candidates understood the impact of systemic inequities. Well-known and embedded approaches such as mentoring programs, employer resource groups or affinity groups were often mentioned as a positive approach to ensure retention and to create a sense of belonging. Moreover, they pointed out the role of psychological safety in speaking out against racist behavior as one important aspect for retention. To encourage leadership staff to promote equity and anti-racism, one hospital introduced bonuses for leadership staff if they met equity goals such as increasing leadership diversity and meeting equity goals for patients. Those bonuses were part of an executive compensation scheme and led to a 2.5% increase in minority faculty membership. Other organizations introduced self-imposed goals such as increasing diversity of leadership. Hospitals established a Diversity, Equity and Inclusion [DEI] council to monitor progress on diversity in leadership and promoted protocols established to create transparency and check them for an equity lens. Creating accountability, especially for leadership staff, was mentioned as one important aspect, as was retraining or removing leaders whose values and behavior did not match = the values of the organization. These approaches created an alignment between the organization’s goals and the practices: “I don’t think policies drive an organization, but accountability towards the policies do” [HEQ, IP 9].

### 3.3. Address Racial Disparities and Other Social Drivers of Health

Hospitals have started to provide grants to their divisions to conduct studies on racial inequities in health status and implement tailored interventions to address them. Establishing health equity dashboards and developing reporting tools that track disparities in patients, as well as employees, has become a common practice in many hospitals. Interviewees reported electronic health record prompts as another way to address [racial] bias in clinical care. These prompts would appear during referral to remind health care professionals of systemic inequities in health care and give the professionals a “nudge to pause and reflect” on their referral decision in order to disrupt implicit bias. An essential way to address racial disparities identified by all interviewees was to strengthen community engagement and engage in so-called ‘listening sessions’, ‘community forums’ and ‘townhalls’ that have community advisory boards and develop recommendations and standards for clinical care [such as pain management protocols] with the communities they partner with in order to rebuild trust. Moreover, interviewees pointed out the importance of data collection and analyzing the data through a [racial] equity lens. Based on the data, they introduced performance measures to prioritize the different areas of action and close gaps between the health outcomes and access to health care of different populations. In this context, one interviewee pointed out that anti-racist work was not just about numbers: “Numbers help us keep track of our work. Are we doing good? Are we doing bad? But numbers are not what creates an anti-racist institution. It’s the will of the people and leaders to say this is how we need to be, how we need to show up and that commitment to all points in time and holding others accountable and holding each other accountable within our institutions” [IP 4, PA].

Many interviewees had an intersectional perspective and emphasized that racial disparities were heavily interconnected with other forms of discrimination, practices of exclusion, such as incarceration and residential segregation, and social drivers of health, such as access to food, housing and transportation. Screening for these possible barriers and including this information in the electronic patient record, giving out healthy food, creating better neighborhoods and being culturally responsive to all patients were mentioned as broader actions that health care organizations needed to consider and or implement in order to successfully address racism. Ultimately, they pointed out that anti-racism work within an organization was quality, safety and improvement work, as this was considered something “health care organizations have a commitment to” [IP 3, CSHD], and these values served as “concrete entry points for incorporating equity into their work” [IP 5, PA].

### 3.4. Racist Behavior

Interviewees agreed that repeated and intentional racist behavior from staff or patients needed to be addressed and have sanctions. The practices identified to achieve that aim were different. One hospital developed a code of conduct for patients that foresees a step-by-step process to remind patients of their obligation to respect staff: “remind the workforce that they deserve protection from patients who call them names, swears and gives us the right to flag them and potentially terminate the treatment”. Other hospitals had a DEI SWAT team that trained leaders on how to speak with patients regarding DEI complaints and “then also kind of creates a conference to assess, A, what does this patient need? What are some of the barriers that they may be feeling that are expressing themselves through this inappropriate behaviour B, to reinforce what our standard of care is and what our expectations are. And, C, to evaluate long-term care” [HEQ, IP 9]. Moreover, so called ‘employer resource’ or ‘affinity groups’ were also considered as a space to talk about racist experiences from patients and provide information about the support services within an organization. In order to protect patients from racism and discrimination, one hospital had a civil rights office as part of their hospital system to ensure equitable care was delivered. Another interviewee reported their hospital had introduced a peer group that would meet with the employee whose behavior was discriminating with the aim of creating a different sense of understanding and accountability amongst the peer group. Another approach was to appoint [volunteer] persons who noticed racist behavior and “called people in” instead of “calling them out” publicly [IP 3, CSHD] in order to interrupt racist behavior. In this context they also mentioned that this duty should most importantly be performed by all senior staff and people in power in organizations and not only by racialized employees in order to prevent mental and emotional drain.

### 3.5. Challenges and Strategies in Anti-Racism Work

Interviewees agreed that the Black Lives Matter movement and the COVID pandemic created an increased momentum for anti-racist change in health care, and many stakeholders were receptive of change. They pointed out that anti-racist work in health care organizations requires strategic planning and should not only be dependent on leadership but a collective responsibility and part of the foundational work of an organization through mission statements, equity standards and goals. Interviewees saw that one component of their work was to create policy and training spaces to increase awareness of racism in health care. This would be performed by delivering [racial justice] training, internal lectures on anti-racist clinical care and workplace events to illuminate how white supremacy and racism show up at the workplace and providing strategies and messaging to move toward racial healing or health equity rounds with guidance and commitment from senior leaders. By creating these spaces, they hoped to normalize anti-racism work and increase understanding of systemic and institutional racism in health care. In doing so, they hoped to change the “dominant mind-set” [IP 2, CSHD] in health care from putting the blame on personal behavior to understanding the structural causes of [health] inequities and thereby encouraging health professionals to “advance anti-racist equity principles in the workplace” [CSHD, IP 3].

Interviewees referred to the national emergence of backlash against anti-racism and DEI efforts as a challenge and stated that the current policy environment was not favorable. Protests and threats against people working in this field were seen as a threat to advancing racial equity itself but also a strategy to derail advancement. In this context, leadership support is crucial in times when “society is making it harder for them to advance this issue” [RI, IP 6], and leadership support was mentioned as one strategy to be able to do the work. A strategy identified to advance anti-racist practices was to create surrounding conditions that “make it tough to say no and easy to say yes” [HEQ, IP 10] for the workforce and leadership within an organization and go through “consensus processes” [IP 9, HEQ] to make sure to include everyone in the process. Moreover, tying anti-racism work to quality and safety in patient care led to a strategic advancement of anti-racism work. Interviewees pointed out that whenever people needed to be convinced of the importance of this topic, it was helpful to have ‘equity champions’ that people could trust and identify with. Moreover, it was important to acknowledge the work and give out equity awards to projects and departments that were progressing in achieving equity goals.

Interviewees criticized the end of certain policies, such as affirmative action, which was seen as further exacerbating the existing lack of representation of racialized professionals in health care. Broader systemic inequities [e.g., racism in the housing or financing sector], a lack of resources in health care, siloed work and the discomfort while talking about privilege in this context were also identified as challenges.

## 4. Discussion

This research aimed to provide insights into much needed solutions and practices to address racism in health care. This study was conducted prior to the 2025 political shift in the US. It reflects a momentum when DEI and anti-racist approaches were supported in many health care organizations. Today those same practices may lead to a restriction of funding and severe backlash. In light of this policy shift and the restrictions of funding, the practices identified in this research remain essential for advancing equitable care. They underscore the need for evidence-based approaches that can withstand political cycles, protect the rights of marginalized patients and integrate equity principles into broader frameworks such as patient safety, quality improvement and workforce wellbeing. Rather than diminishing their relevance, the changing landscape highlights the urgency of safeguarding and embedding these practices into the core functions of health care organizations so that they endure, regardless of shifts in federal priorities, and are aligned with the understanding of racism as a systemic and structural part of health care.

Rarely are inner organizational practices published in scientific journals, and without bridging this gap between research and practice, there will continue to be a lack of easily accessible and usable examples of anti-racist practices in health care [4]. This study aimed to address this gap and has contributed to making visible organizational solutions and practices that can be adopted and applied by other organizations. In this study, the broad definition of anti-racism was operationalized as a set of organizational-level practices. The operationalization allowed us to translate the concept of anti-racist practices into a concrete mechanism of organizational theory. Accordingly, anti-racism is measured through evidence of policies and actions focused on (1) data collection and transparency, (2) normative integration of anti-racist standards, (3) development of proactive organizational policies, (4) establishment of accountability and consequence procedures and (5) the creation of culturally safe spaces.

This study has made clear that every health care organization can use their influence to address racism and use a broad range of practices to do so. The examples illustrate how organizational change is rarely driven by single actions but emerges across multiple levels of the health care ecosystem and within institutions. This is a finding consistent with Kotter’s change model. Health care organizations effectively act as guiding coalitions that can create enabling conditions for hospitals to adopt and sustain anti-racist practices, for example, targeted career pathway approaches and inclusive strategies to create feelings of belonging. Moreover, through creating positive incentives for meeting equity goals, an organization can increase motivation and awareness for the importance of equity that in the above-described case led to a 2.5% increase in minority faculty. This was also described in another major hospital system in the US, within their health equity and anti-racism strategy: “executives are held accountable for organizational performance on diversity and equity through an institutional diversity index linked to executive pay” [14]. This action directly aligns with Kotter’s imperative to generate short-term wins and institutionalize new approaches [10]. While this could indicate progress, it may be insufficient to make a significant impact on overall workforce diversity. Even with these efforts, in terms of increasing workforce diversity and leadership of underrepresented groups, there is a lack of representation. This is despite studies showing that workforce diversity can enhance trust between providers and patients, as well as improve health outcomes [15]. “Just 14% of hospital board members and 9% of CEOs are minorities, according to the most recent study by the American Hospital Association’s Institute for Diversity and Health Equity—a number that has remained unchanged since 2013. At the same time, BIPOC [Black, Indigenous and People of Color] constitute about a third of hospitals’ patients, and that figure is growing” [8]. Benchmarking tools such as the Medicaid Primary Care Workforce Tracker [16] or the Healthcare Equality Index [17] can create visibility in terms of workforce shortage, underrepresentation and disparities between states as well as health care organizations and encourage an impetus to change the status quo. None of the approaches described seemed to allude to one of the major underlying challenges with regard to anti-racist accountability in early education and admission [18]. However, this could support creating equitable pathways and prepare racialized groups for a career in health care, which would ultimately lead to an increase in the pool of people who could be hired. This would also align with structural anti-racism perspectives that address upstream barriers to produce sustainable improvements. The introduction of equity goals through the accreditation organizations, required equity metrics through federal agencies and the community health needs assessment were seen as useful policy levers to promote health equity within their organization [19,20]. From an organizational change perspective, tying equity goals to incentives and evaluation systems is a mechanism for embedding new norms into routines. These kind of external mandates can be vital in creating a sense of urgency [10]. While these requirements can help standardize equity practices and create tools for transparency and accountability within each hospital, organizations need to be prepared to address implementation challenges such as data collection, resistance from within and the scarcity of resources in order to make sure that their strategy to meet the equity goals is sustainable.

Moreover, it may not be enough to systematically address racial disparities in health care that result from a continuous history of racism and exclusion in health care. Addressing disparities through dashboards will require data collection and monitoring first, in order to define a baseline and identify gaps and limitations, which is a challenge itself. In the US, more “than 87 percent of hospitals report collecting race and ethnicity data, and 90 percent report collecting data on primary language” [21]. At the same time, “fewer than one in five hospitals that collected these data used them for any of these purposes such as the assessment of quality of care, the utilization of health services or health outcomes” [22]. This shows that most health care organizations in the US do not systematically collect, disaggregate and analyze data in order to improve racial inequities. Improved data collection, analysis and management would also assist in addressing the misclassification and data erasure of Indigenous people [3], who have often been “misracialized as other racial or ethnic identities in population health research” leading to “underestimation of Indigenous-specific mortality and health metrics, and subsequently, inadequate resource allocation” [23]. In this context, it is important to critically assess the data to benefit the health of Indigenous people and be aware of the fact that data collection alone is insufficient unless it is coupled with organizational and systemic changes in policy and resource allocation. Scholars have called for a re-evaluation of how data are collected, analyzed, and used, particularly when it comes to Indigenous and other minoritized communities. Thus, it is crucial to not only collect and analyze data but to apply them in ways that challenge and address systemic racism within health care organizations and pay attention to “community engagement, research oversight, and capacity building” [24]. But, collecting and analyzing data is just the first step to detect racial inequities within an organization. It is crucial to use the data to implement tailored interventions that further reduce racial disparities. One such example is the anti-racism campaign of one hospital system [25] to reduce racial disparities, which includes interventions such as developing and delivering multilingual correspondence, hiring bilingual community health workers, improving race-based data collection and establishing restorative justice circles to promote racial healing [26]. Moreover, introducing nudges, such as electronic prompts in electronic patient record systems is a relatively new approach that disrupts everyday work by reminding us of the implicit bias in medicine that can cause harm to patients. As this was a pilot program, no evidence of the effectiveness of these prompts is available at this stage. Nevertheless, the example highlights that anti-racist work in organizations is quality, improvement and safety work.

Thus, in order to address racial disparities in health care, an anti-racist patient-centered model of care should be favored that emphasizes individualized care while considering social, political and environmental drivers of health. This can also be achieved by genuine and ongoing community engagement. As the experts in this study pointed out, engagement is a way of making sure that health care services are responsive to communities needs and also an acknowledgement of the wisdom and knowledge that exists within the communities. Research suggests that centering communities and people most affected by systemic racism can be a significant way to empower people and dismantle racism [3].

While there was consensus that racist behavior from staff and patients should have consequences, it remained unclear in most cases in what manner these consequences were communicated, by whom and how. The absence of consistent enforcement procedures illustrates how structural racism operates through organizational silence and ambiguity. This reveals a gap in the implementation of consistently enforced accountability mechanism for racist behavior. In alignment with structural anti-racism and organizational change approaches, clarifying responsibility and supporting leaders in enforcing policies are necessary practices. One guideline [27] demonstrates a process guideline on how to deal with discriminatory behavior from patients. It outlines the ethical implications of that decision-making process and shows the need for an interprofessional and interdisciplinary team to make this decision. At the same time, it should be clear that patients, as well as staff, need to be protected from discrimination, as it affects their health [28] and guidelines could give employees orientation and a framework to better respond as bystanders to racist behaviors and/or be protected from racist behavior. This seems especially important considering that up to 40 percent of primary care physicians report “that the health system treats people differently based on their racial or ethnic background” [29]. Moreover, this amplifies the need to have spaces, such as employer resource groups, to process these experiences and find likeminded people. Listening sessions and townhalls could be one way to improve health care services based on including community voices. But, more than that, it is necessary for hospitals to incorporate this feedback and provide space for patients that have experienced racism in health care and may have lost trust. Empowerment spaces, healing circles and mental health support for patients who experience racism in health care are essential in order to promote community-oriented health promotion practices [30], increase patients’ agency in advocating for their rights and prevent weathering [31] and health-related consequences of the experiences of racism. In general, progress seems to be incremental, although “racial health disparities are not inevitable and can be eliminated with the appropriate intervention” [32]. The role of civil rights offices was seen as one way to address these issues, and although understaffing and a scarcity of resources are criticized as two hurdles to advancing equity, the bigger challenge is to enable the federal government to act and seriously invest in addressing and eliminating structural health care system inequities [33]. It is disconcerting that this reality persists despite the high rates of discrimination experienced by patients, noted in a recent report [34].

All experts mentioned the intersectionality of the different forms of discrimination with other social, political and environmental drivers of health as broader factors within their anti-racism work. They pointed out that the political climate, the backlash against DEI and anti-racism efforts are a major distraction and [personal] threat to achieving health equity. Even within health care organizations, there are efforts to reverse DEI policies [35]. Although “Diversity, Equity, and Inclusion [DEI] is the organizational approach to ensure fair treatment and welcomed participation of all groups” [36], there have been policy changes in multiple states that require public and private organizations to eliminate DEI vocabulary and trainings if they want to obtain state funding. These developments make it even more important to protect the work and build strong alliances within the health care ecosystem with the support of leadership, as one leader in the field recently reminded us: “it is important to remember that eliminating DEI efforts negatively affects not only Black people, but all racially and religiously marginalized groups, Indigenous people, women, non-Christians, people with disabilities, and LGBTQ+ people” [37]. Ultimately, in order to effectively improve health outcomes, there is a need to look beyond the health care system as “structural racism refers to the totality of ways in which societies foster racial discrimination through mutually reinforcing systems of housing, education, employment, earnings, benefits, credit, media, health care, and criminal justice” [38].

## 5. Conclusions

The identified practices demonstrate that organizations have a wide range of possibilities to embed equity into daily operations and accountability procedures. Anchored in a structural understanding of racism and guided by the principles of organizational change, the results highlight how health care organizations can be places of transformation when equity and anti-racism become part of the operational logic rather than optional ideas. This study contributes to closing a know–do gap and facilitates much needed discussions about possible anti-racism actions and practices within organizations. Given the political shift in the US, it is crucial to acknowledge that on the one hand, the identified anti-racist practices may now put organizations in jeopardy in terms of political and funding conditions. One the other hand, this highlights the importance of protecting and institutionalizing these practices and recognizing them as a core component of quality, safety and professional integrity.

### Strengths and Limitations

While this study was not designed to identify the effectiveness of these practices, it provides promising strategies for embedding anti-racist practices within organizations to a wider audience. It is important to note the limited evidence supporting the effectiveness of the identified practices. Another limitation is the small sample size that excluded certain stakeholders of the health care ecosystem, such as insurance companies, as well as federal- and state-level organizations, and only focused on practices in the US. Moreover, we note that due to not collecting demographic data, we cannot clearly determine how positionality may have shaped perspectives. Given the study’s exploratory nature and the additional use of secondary data, we prioritized capturing a diverse range of perspectives rather than aiming for full data saturation, which is a limitation. As racism is a global problem, future research should look at organizations globally to identify anti-racist practices in organizations and identify potential recommendations. Additionally, comparative research across different national contexts—such as the US and countries where race data are not systematically collected—could provide valuable insights into how structural and policy differences shape anti-racism efforts in health care organizations. Moreover, future research should also examine the enforcement of sanctions and repercussions to analyze the effectiveness of these policies. For this study, a possible selection and confirmation bias amongst the interviewees cannot be eliminated. In fact, all of the interviewees in this study work in urban hospitals systems and health care organizations and may not reflect the reality of rural hospitals. We also understand that the examples highlighted in this study may not fully reflect the breadth of systemic and institutional racism within health care organizations. Nevertheless, interpreting these findings through a structural anti-racism framework allows us to see such absences not merely as data limitations, but as evidence of the ways in which organizational practices continue to reproduce inequities by omission. Lastly, this study did not focus on universities and medical and public health schools that may have experiences that could significantly contribute to addressing racism through teaching and training the next generation of health care professionals [38]. In order to increase transparency and reliability, this study followed the reporting guidelines for qualitative research.

## Data Availability

The data presented in this study are available on request from the corresponding author due to the sensitive nature of the material.

## Supplementary Materials

The following supporting information can be downloaded at: https://www.mdpi.com/article/10.3390/ijerph22111641/s1. Table S1: overview of methods, File S1: participant demographics.
