# Supplementary material for: Anti-Racist Practices in Health Care Organizations—A Qualitative Analysis
Source: Int J Environ Res Public Health. 2025 Oct 28;22(11):1641. doi: 10.3390/ijerph22111641 (PMC12652409; doi:10.3390/ijerph22111641)
Supplement: Supplementary file 1 [file ijerph-22-01641-s001.zip › ijerph-3849297-Supplementary File S1.pdf]

COREQ (COnsolidated criteria for REporting Qualitative research) Checklist

A checklist of items that should be included in reports of qualitative research. You must report the page number in your manuscript where you consider each of the items listed in this checklist. If you have not included this information, either revise your manuscript accordingly before submitting or note N/A.

Topic	Item No.	Guide Questions/Description	Reported on Page No.
**Domain 1: Research team and reflexivity**			
Personal characteristics			
Interviewer/facilitator	1	Which author/s conducted the interview or focus group?	3 (135)
Credentials	2	What were the researcher's credentials? E.g. PhD, MD	3 (135)
Occupation	3	What was their occupation at the time of the study?	3 (135)
Gender	4	Was the researcher male or female?	3 (135)
Experience and training	5	What experience or training did the researcher have?	3 (136)
Relationship with participants			
Relationship established	6	Was a relationship established prior to study commencement?	3 (125)
Participant knowledge of the interviewer	7	What did the participants know about the researcher? e.g. personal goals, reasons for doing the research	study inf.
Interviewer characteristics	8	What characteristics were reported about the inter viewer/facilitator? e.g. Bias, assumptions, reasons and interests in the research topic	study inf.
**Domain 2: Study design**			
Theoretical framework			
Methodological orientation and Theory	9	What methodological orientation was stated to underpin the study? e.g. grounded theory, discourse analysis, ethnography, phenomenology, content analysis	3/4
Participant selection			
Sampling	10	How were participants selected? e.g. purposive, convenience, consecutive, snowball	3/4 (138/148)
Method of approach	11	How were participants approached? e.g. face-to-face, telephone, mail, email	3/4
Sample size	12	How many participants were in the study?	3 (127)
Non-participation	13	How many people refused to participate or dropped out? Reasons?	3 (128)
Setting			
Setting of data collection	14	Where was the data collected? e.g. home, clinic, workplace	3 (133)
Presence of non-participants	15	Was anyone else present besides the participants and researchers?	3 (133)
Description of sample	16	What are the important characteristics of the sample? e.g. demographic data, date	4 (168)
Data collection			
Interview guide	17	Were questions, prompts, guides provided by the authors? Was it pilot tested?	3 (116)
Repeat interviews	18	Were repeat inter views carried out? If yes, how many?	study inf./N.A
Audio/visual recording	19	Did the research use audio or visual recording to collect the data?	3 (133)
Field notes	20	Were field notes made during and/or after the inter view or focus group?	3 (133)
Duration	21	What was the duration of the inter views or focus group?	3 (124)
Data saturation	22	Was data saturation discussed?	11 (490)
Transcripts returned	23	Were transcripts returned to participants for comment and/or	study inf.

Topic	Item No.	Guide Questions/Description	Reported on Page No.
		correction?	
**Domain 3: analysis and findings**			
Data analysis			
Number of data coders	24	How many data coders coded the data?	4 (154)
Description of the coding tree	25	Did authors provide a description of the coding tree?	Suppl.mat.
Derivation of themes	26	Were themes identified in advance or derived from the data?	4 (155)
Software	27	What software, if applicable, was used to manage the data?	4 (152)
Participant checking	28	Did participants provide feedback on the findings?	N.A
Reporting			
Quotations presented	29	Were participant quotations presented to illustrate the themes/findings? Was each quotation identified? e.g. participant number	4-8
Data and findings consistent	30	Was there consistency between the data presented and the findings?	4-8
Clarity of major themes	31	Were major themes clearly presented in the findings?	4-8
Clarity of minor themes	32	Is there a description of diverse cases or discussion of minor themes?	4-8

Developed from: Tong A, Sainsbury P, Craig J. Consolidated criteria for reporting qualitative research (COREQ): a 32-item checklist for interviews and focus groups. *International Journal for Quality in Health Care*. 2007. Volume 19, Number 6: pp. 349 – 357

Once you have completed this checklist, please save a copy and upload it as part of your submission. DO NOT include this checklist as part of the main manuscript document. It must be uploaded as a separate file.

Appendix A5. Characteristics of interview participants

	Interview Participants	
	**N**	**%**
**Level of Responsibility of Equity Position**		
Individual hospital	10	56
Several hospitals	0	0
Health system	8	44
**Length of Time in Equity Position**		
<6mo	6	33
6mo to <1yr	2	11
1yr to <2yr	5	28
2yrs to <5yrs	3	17
5 or more yrs	2	11
**Gender Identity**		
Male	6	33
Female	12	67
Non-binary or transgender	0	0
None of the above, or preferred not to answer	0	0
**Race**		
White	9	50
Black or African American	6	33
American Indian	0	0
Alaskan Native	0	0
Other Pacific Islander	0	0
Asian	3	17
Some Other Race	0	0
Prefer not to answer	0	0
>1 race	1	5
**Hispanic or Latino**		
Yes	1	5
No	17	95

Permission to Take Part in a Human Research Study

Protocol Title: Anti-Racist Interventions in Health Care
Principal Investigator: Sidra Khan Gokkaya<br>Faculty Advisor (if PI is a student): David R. Williams
Description of Study Population: Experts
Version Date: 01/08/2024

Key Information

The following is a short summary of this study to help you decide whether or not to participate.

Why am I being invited to take part in a research study?

You are invited to participate in a research study exploring systemic racism and anti-racist interventions in the context of healthcare. The purpose of this study is to identify and understand systemic changes that can address racism within healthcare systems, as well as to explore the effectiveness of anti-racist interventions. The study aims to gain insights from experts in healthcare.

What should I know about a research study?

- Someone will explain this research study to you.
- Whether or not you take part is up to you.
- You can choose not to take part.
- You can agree to take part and later change your mind.
- Your decision will not be held against you.
- You may discuss your decision with your family, your friends and/or your doctor.
- You can ask all the questions you want before you decide.

Why is this research being done?

The primary objectives of this research are to:

- Investigate how systemic change in healthcare, concerning anti-racism, manifests.
- Examine the types of interventions implemented in healthcare systems to address racism.
- Identify the prerequisites for implementing anti-racist interventions in healthcare.

How long will I take part in this research?

If you agree to participate, you will be asked to take part in a confidential virtual interview. The interview will be conducted by the principal investigator. The interview will involve open-ended questions related to systemic racism, anti-racist interventions, and organizational challenges and resources. The interview is expected to last approximately 60 minutes.

What will I be asked to do?

If you agree to participate, you will be asked to take part in a confidential virtual interview and answer open-ended questions about your experiences and insights into anti-racist interventions in health care.

Is there any way being in this study could be bad for me?

Participation in this study involves minimal risk. The information provided during the interview may evoke personal or professional reflections on systemic racism in healthcare. However, efforts will be made to ensure a respectful and supportive environment during the interview process.

Will being in this study help me in any way?

We cannot promise any benefits to you or others from your taking part in this research. However, the potential benefits of participating include contributing to the understanding of systemic changes and anti-racist interventions in healthcare, which may inform future efforts to combat racism in the healthcare sector.

What happens if I do not want to be in this research?

Participation in research is voluntary. You can decide to participate, not to participate, or stop participation at any time without penalty or loss of benefits to which you are otherwise entitled.

Statement of Consent

I have read the information in this consent form including risks and possible benefits. All my questions about the research have been answered to my satisfaction. I understand that I am free to withdraw at any time without penalty or loss of benefits to which I am otherwise entitled. I understand that if I am returning this form electronically or remotely that all pages must be sent back to the researchers.

I consent to participate in the study.

Omit the signature section below if you are requesting a waiver of written documentation of consent.

SIGNATURE

Your signature below indicates your permission to take part in this research

Permission to Take Part in a Human Research Study

Name of participant

Signature of participant | Date

Signature of person obtaining consent | Date

Printed name of person obtaining consent
