# Supplementary material for: Anti-Racist Practices in Health Care Organizations—A Qualitative Analysis
Source: Int J Environ Res Public Health. 2025 Oct 28;22(11):1641. doi: 10.3390/ijerph22111641 (PMC12652409; doi:10.3390/ijerph22111641)
Supplement: Supplementary file 1 [file ijerph-22-01641-s001.zip › ijerph-3849297-Supplementary Table S1.pdf]

Table S1. Overview of methods.

Dataset	Primary data set	Secondary data set
Origin	Expert interviews conducted by first author	Study on Health Equity Officers
Participants	N=11	N=26
Participant characteristics	Individual demographic data was not collected in accordance with the institutional board review	Overview of participant characteristics is available in the supplementary material
Analysis	Qualitative Content Analysis (MAXQDA)	Qualitative Content Analysis (MAXQDA)
Transcripts analyzed for this study	37	
